# P-416. The Unexpected Costs of Cutting Costs

**DOI:** 10.1093/ofid/ofae631.617

**Published:** 2025-01-29

**Authors:** Palak Patel, R Marrs, Molly Steele, Vera Chu, Patricia D Zuccaro, Allison H Bartlett, Emily Landon

**Affiliations:** The University of Chicago, Chicago, Illinois; University of Chicago Medicine, Chicago, Illinois; University of Chicago, Chicago, Illinois; UChicago Medicine, Chicago, Illinois; The University of Chicago Medicine, Chicago, Illinois; University of Chicago Comer Children's Hospital, Chicago, IL

## Abstract

**Background:**

Hand Hygiene (HH) is the cornerstone of preventing healthcare-associated infections, however maintaining compliance is challenging especially when staffing changes. Here we show HH performance as measured by an automated monitoring system before and after a reduction in force (RIF) affecting unit-based nursing leadership.

**Figure:**
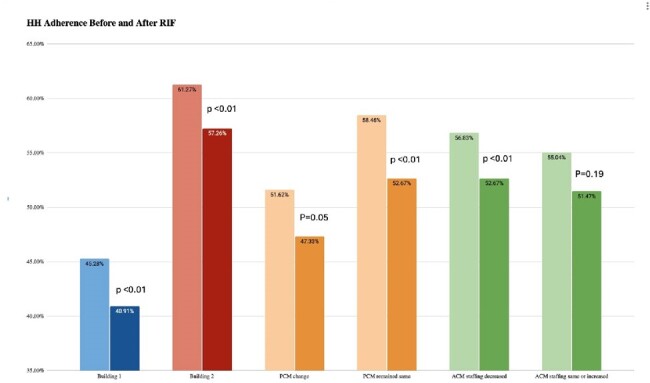

**Methods:**

University of Chicago is a large 800 bed academic medical center with 30 adult inpatient units that monitors HH using the GOJO Smartlink System (Akron, OH) to track entries and exits (denominator) as well as soap and hand sanitizer dispenses (numerator) aggregated over a nursing unit. The adult inpatient units span across two buildings and aggregate compliance data by nursing unit from Jan 1, 2024 through Mar 30, 2024 is included. A 4% RIF that changed unit-based nursing leadership but not direct patient care staff took place on 2/1/2024. The paired t-test was used to compare data from before and after the RIF.

**Results:**

Building 1 had HH rate 45.28% in January before the RIF and 40.91% after the RIF (p< 0.01). For building 2, HH before the RIF was 61.27% before and 57.26% after the RIF (p< 0.01). 10 units had a change in nursing manager (PCM) and 20 units had a 50% reduction in assistant manager (ACM) staffing. In univariate analysis, both units with a change in PCM (51.62% to 47.33%, p=0.05) and those without a change in PCM (58.46% to 54.78%, p< 0.01) had statistically significant decreases in HH. Units with a decrease in ACM staffing also had a significant decrease in HH (56.46% to 52.67%, p< 0.01) but those who kept their ACM staffing did not (55.04% to 51.47%, p=0.19).

**Conclusion:**

Overall, HH performance decreased significantly after the RIF despite not affecting any direct patient care roles. A decrease in staffing of the Assistant Care Manager role may have a larger impact on HH adherence than PCM role.

**Disclosures:**

**Allison H. Bartlett, MD, MS**, CVS/Caremark: Advisor/Consultant

